# Using Synthetic Glycans to Investigate Anti‐Glycan Antibodies and Explore Their Medical Potential

**DOI:** 10.1002/anie.7795967

**Published:** 2026-07-27

**Authors:** Fabienne Weber, Aina Valenti, Jiří Ledvinka, Oren Moscovitz, Peter H. Seeberger

**Affiliations:** ^1^ Department of Biomolecular Systems Max Planck Institute of Colloids and Interfaces Potsdam Germany; ^2^ Institute of Chemistry and Biochemistry Freie Universität Berlin Berlin Germany; ^3^ Scojen Institute for Synthetic Biology Dina Recanati School of Medicine Reichman University Herzliya Israel

**Keywords:** anti‐glycan‐antibodies, monoclonal antibodies, synthetic glycans, vaccines

## Abstract

The dynamic glycan layer that surrounds human and microbial cells plays an essential role in the immune system's capacity to maintain immune tolerance and equilibrium, fight pathogens and cancer. Endogenous anti‐glycan antibodies are an indispensable part of immune surveillance, identifying abnormal microbial glycans, viral glycoproteins, and altered tumor‐associated carbohydrate antigens. These glycans serve in turn as attractive targets for passive and active immunization strategies in a range of human diseases. The enormous complexity and structural diversity of glycans has made it very difficult to unlock their full biomedical potential. Recent advances in glycan synthesis, including automated solid‐phase assembly, chemoenzymatic strategies, and one‐pot approaches, enabled unprecedented access to well‐defined, homogenous structures. Hence, synthetic glycans of increasing structural complexity pave the way for a range of applications, from profiling endogenous antibody repertoires for biomarker discovery to therapeutic antibody development and vaccine design. Here, we summarize the strategies to utilize synthetic glycans for antibody development and their application in basic research and translational medicine. We outline how anti‐glycan antibodies are utilized for diagnostic and therapeutic purposes, while emphasizing the power of synthetic glycans and highlighting their potential in personalized medicine.

## Graphical abstract

Anti‐glycan antibodies are essential in health and disease. Access to novel glycan structures paves the way for progress in antibody profiling for biomarker discovery, antibody development, and vaccine design. We summarize the strategies to synthesize and utilize synthetic glycans for the development and application of anti‐glycan antibodies in basic and translational research.

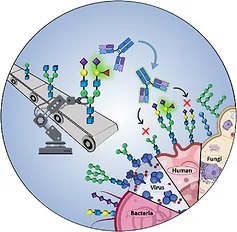

## 1 Introduction

**FIGURE 1:**
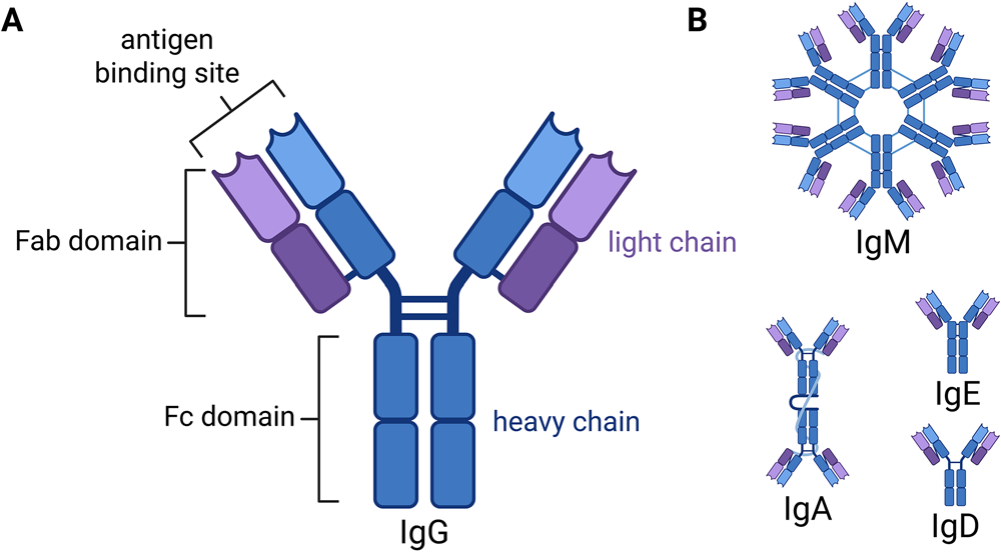
Structure of the different antibody types. IgG; immunoglobulin G; IgM; IgA; IgE, and IgD; Fab, fragment antigen binding; Fc, fragment crystallizable.

The mammalian immune system has evolved advanced mechanisms to distinguish between self and foreign. In response to abnormal, foreign or altered structures, so‐called antigens, specialized immune cells produce large amounts of antibodies (Abs), comprising around 15%–20% of the total protein pool in blood serum [1, 2]. Humans produce a large and diverse range of Abs that protect against a multitude of pathogens and foreign substances. Human Abs are Y‐shaped heterodimers of heavy and light chains linked by disulfide bonds, containing fragment antigen binding (Fab) domains that bind the antigen and fragment crystallizable (Fc) domains that determine the Ab isotype and function. Five isotypes of antibodies with different effector functions are distinguished: immunoglobulin G (IgG), IgM, IgA, IgE, and IgD (Figure 1) [3, 4]. IgGs are monomeric, generally more stable and specific than other isotypes, and represent the most abundant isotype in blood or serum. IgMs can be found in both pentameric and hexameric forms, characterized by low affinity but high avidity. IgAs are predominantly dimeric and are the most common isotype on mucosal surfaces [4]. The less abundant isotypes are IgE, primarily involved in allergic reactions and responses to parasites, and IgD, which may be involved in immune regulation and B cell activation [5]. Abs are being produced upon stimulation by endogenous or exogenous antigens. Fab‐mediated binding triggers Fc‐mediated defense mechanisms, including neutralization, phagocytosis, antibody‐dependent cellular cytotoxicity, or killing via complement activation [5]. Sometimes, antibody‐mediated responses to pathogenic antigens exacerbate the disease by antibody‐dependent enhancement [6]. Immune tolerance failure and the inability of the immune system to distinguish between self and foreign antigens generate autoantibodies that target self‐epitopes and lead to the development of autoimmune diseases [7].

Glycans are universally present in nature and essential for life [8]. The glycocalyx, a dense layer of diverse glycans coupled to proteins, lipids, nucleotides, and additional glycans surround human, bacterial, and pathogen cells [9, 10]. There are 10 common monosaccharide building blocks (BB) forming the human glycocalyx: glucose (Glc), galactose (Gal), *N*‐acetylglucosamine (GlcNAc), *N*‐acetylgalactosamine (GalNAc), glucuronic acid (GlcA), iduronic acid (IdoA), xylose (Xyl), mannose (Man), fucose (Fuc), and the sialic acid *N*‐acetylneuraminic acid (Neu5Ac) (Table 1) [11]. Non‐human mammals also contain the sialic acid *N*‐glycolylneuraminic acid (Neu5Gc) that humans can incorporate through diet [12]. Complex glycosylation machineries create structural variability across species. These different glycans are recognized by Abs and carbohydrate‐binding proteins (lectins) to distinguish self and non‐self [13, 14]. Glycoproteins carry *N*‐glycans attached to asparagine (Asn) and *O*‐glycans that are connected to serine (Ser) or threonine (Thr) residues [15]. The function of Abs that are also glycoproteins containing conserved Fc *N*‐glycans depends on this glycosylation. Other glycoconjugates include glycosaminoglycan, glycosphingolipids [16], glycosylphosphatidylinositol (GPI)‐anchored proteins [15], and ribonucleic acid (RNA)‐coupled glycans [17].

**TABLE 1:** Most common monosaccharides in mammals.

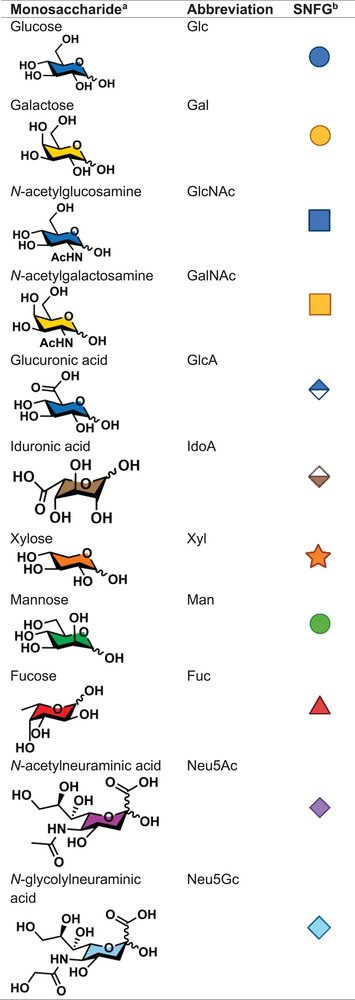

^a^Wavy bonds depict unspecified configuration at the anomeric center.

^b^SNFG, Symbol Nomenclature for Glycans [18].

Microbial glycans are more complex than human glycans as they are composed of more monosaccharides, linked and branched differently, and contain different modifications. These glycans play a significant role in pathogen colonization, proliferation, and immune evasion. Endogenous anti‐microbial glycan Abs that recognize glycan structures on bacteria, fungi, and parasites, contribute to pathogen recognition by the immune system, control colonization, provide immune protection, and maintain microbiome homeostasis [19]. Bacteria produce hundreds of unique sugar units. Key bacterial glycans include capsular polysaccharides (CPS), lipopolysaccharides (LPS), peptidoglycan, cell wall polysaccharides (CWPS), glycosylated lipoteichoic acid (LTA) and wall teichoic acid (WTA) [20, 21, 22]. Membrane proteins often remain masked under carbohydrate layers, defining species‐specific serotypes (ST) [23]. Bacterial carbohydrates exhibit extreme diversity from trioses to dodecoses [20], with modifications like alkyl, acyl, phosphoryl, and nucleosides [24]. Similarly, structurally complex polysaccharides and glycoproteins compose the fungal cell walls and vary by species. The cell wall of most fungi contains chitin, a homopolymer of GlcNAc, as well as the β‐D‐glucose polysaccharides β‐(1→3)‐glucan and α‐(1→3)‐glucan. Mannans, polymers composed of mannose, are another key structural feature. Some, like *Cryptococcus neoformans* (*C. neoformans*), produce a polysaccharide capsule [25]. The outer layer of *Candida albicans* has GPI‐anchored mannoproteins containing linear *O*‐mannans and highly branched *N*‐mannans. The inner layer is composed of chitin, and β‐(1→3)‐ and β‐(1→6)‐glucans [26, 27]. In contrast to bacterial and fungal glycans, viruses rely on the human glycosylation machinery to generate glycan shields for host colonization, replication, and immune evasion [28].

Endogenous anti‐glycan Abs also target altered mammalian glycans, notably in cancer, where dysregulated glycosylation produces aberrant structures [29]. Tumor‐associated carbohydrate antigens (TACAs) on cancer cells contribute to tumor progression, metastasis, immune evasion, and other hallmarks of cancer [15]. Prominent TACAs such as β1,6 GlcNAc‐branched *N*‐glycans, LacdiNAc (β‐GalNAc‐(1→4)‐GlcNAc disaccharide and derivatives), Thomsen‐nouveau (Tn, cluster of differentiation (CD) 175), Thomsen–Friedenreich (TF), and their sialylated (bearing Neu5Ac) counterparts sTn, sTF, and disyalilated TF (dsTF), are frequently overexpressed in multiple cancers, and often correlate with an immunosuppressed microenvironment [30]. Other sialylated TACAs, such as the Lewis family antigens sialyl‐Lewis^X^ (CD15s) and sialyl‐Lewis^A^ (CA19‐9) are involved in metastasis, while glycosphingolipids, such as the stage‐specific embryonic antigen‐3 (SSEA‐3), Globo‐H, the mono‐ and di‐ sialylated gangliosides GM2, GD2, and GD3, drive cancer cell proliferation, survival, and metastasis in multiple cancer types [15, 31]. Monoclonal and endogenous anti‐TACA Abs are promising tools for cancer diagnostics and therapeutics, with several monoclonal Abs (mAbs) already in early clinical trials [32].

Autoantibodies may arise against self‐glycans leading to autoimmune diseases. Triggers may include glycosylation dysregulation or microbial molecular mimicry of self‐glycans [33, 34, 35]. Such harmful autoantibodies are implicated in multiple sclerosis (MS) [36], anti‐ myelin‐associated glycoprotein (MAG) neuropathy [37], type I diabetes [38], inflammatory bowel disease [39], and ulcerative colitis [40]. Autoantigen targets include gangliosides in MS [41], the human natural killer‐1 (HNK‐1) carbohydrate on myelin anti‐MAG neuropathies [37], and microbial‐like glycans such as mannobioside and chitin in inflammatory bowel diseases [39, 40, 42]. The limited understanding of autoimmune mechanisms complicates diagnosis and treatment. Novel diagnosis and treatment approaches are needed.

Beyond endogenous Abs, active immunization through vaccination, stimulates the immune system to produce antibodies against a specific antigen. Carbohydrate‐based vaccine candidates have demonstrated success in inducing protective Abs, preventing infectious diseases [43, 44, 45], and targeting cancers [46, 47, 48, 49, 50]. Neutral or negatively charged oligo‐ or polysaccharides generally induce a weak T cell‐independent immune response that stimulates B cells to produce low‐affinity antibodies. Thus, they are mostly ineffective as vaccine candidates [51]. In contrast, conjugation to protein carriers introduces T cell‐dependent epitopes, generating long‐lived glycan‐specific memory B cells and high‐affinity Abs [52]. Glycoconjugate vaccines overcome the poor immunogenicity of glycans and are used in licensed vaccines such as Prevnar [53]. Semi‐ and fully‐synthetic glycoconjugate vaccines are being explored with synthetic human and microbial glycans, to improve the efficacy and reproducibility of vaccines to target microbial infections and combat cancer [54, 55].

Conversely, passive administration of therapeutic mAbs provides rapid protection, particularly important in immunocompromised patients. Historically, mAbs against glycan antigens were considered low‐affinity binders; however, glycoconjugate immunization elicits strong IgG responses [56], and high‐affinity mAbs with nanomolar affinities have been developed against both bacterial and cancer antigens [57, 58, 59].

The global mortality due to bacterial and fungal infections associated with antimicrobial resistance (AMR) is rapidly increasing [60, 61] and novel treatment approaches are in high demand. In addition to that, increasing cancer incidence demand protective mAb therapies and novel vaccines [62]. Native glycans used for vaccine and mAb development are often heterogeneous and contain impurities, complicating precise targeting. Synthetic glycans provide well‐defined structures, enabling accurate measurement of endogenous anti‐glycan Abs and the development of theranostic mAbs. Despite significant advances, synthetic glycan production remains limited to a few specialized groups, with most structures being costly and not commercially available, restricting widespread research.

Advances in glycan synthesis providing access to complex structures are essential for preclinical, clinical, and biomedical applications. We summarize recent advances in glycan synthesis and the biomedical applications of synthetic glycans. We focus on anti‐glycan Abs, highlighting synthetic microarrays for biomarker discovery and the use of synthetic glycans for passive and active immunization.

## 2 Synthesis of Glycans

### 2.1 Synthetic Challenges

**FIGURE 2:**
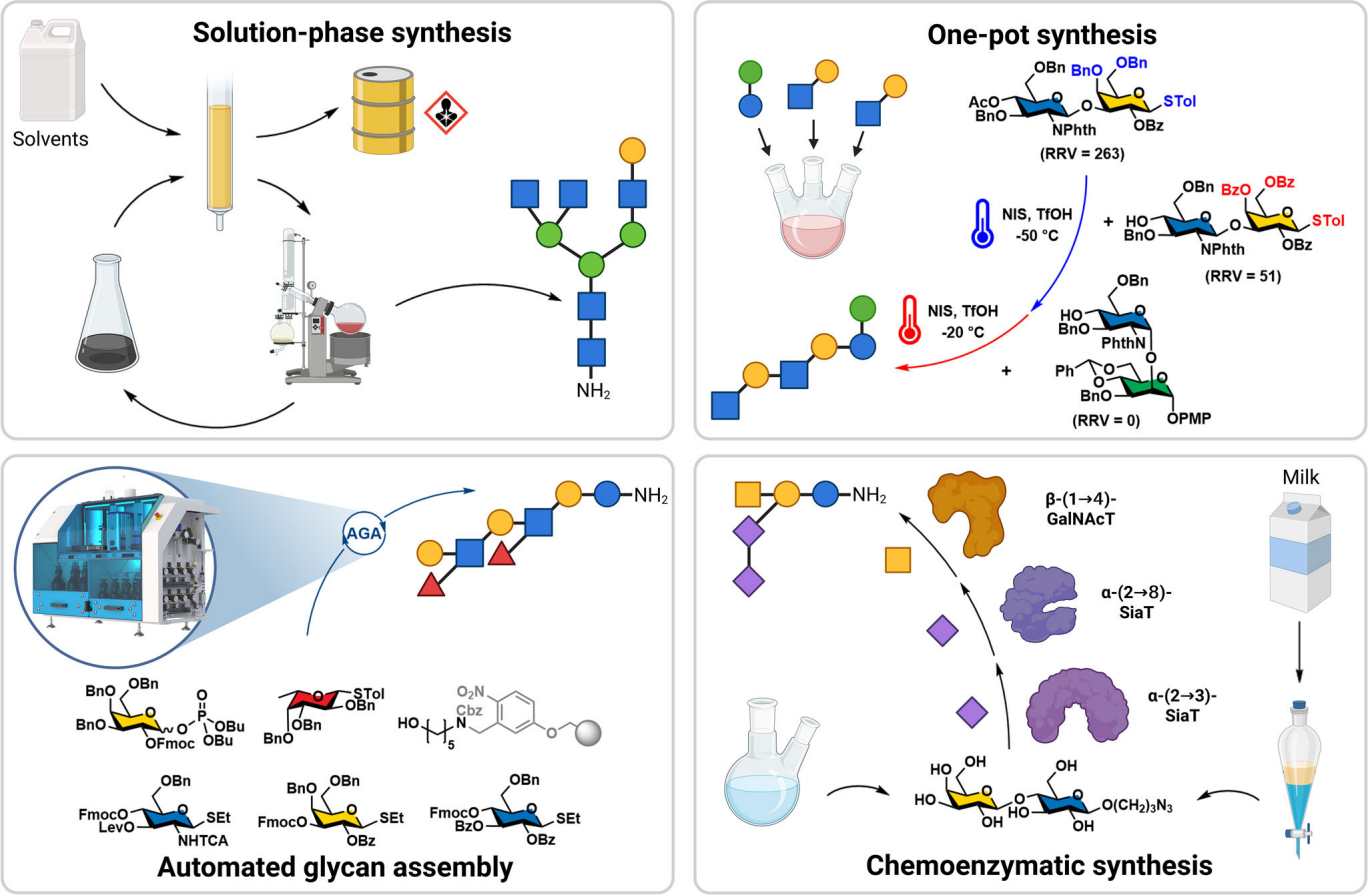
Approaches for glycan synthesis. Glycan synthesis can be achieved by solution‐phase synthesis, one‐pot synthesis in solution, chemoenzymatic synthesis or automated glycan assembly. Ac, acetyl; AGA, automated glycan assembly; Bn, benzyl; Bu, 1‐butyl; Bz, benzoyl; Cbz, benzyloxycarbonyl; Et, ethyl; Fmoc, fluorenylmethyloxycarbonyl; GalNAcT, *N*‐acetylgalactosamintranferase; Lev, levulinoyl; NIS, *N*‐iodosuccinimide; Phth, phthaloyl; PMP, *p*‐methoxyphenyl; RRV, relative reactivity value; SiaT, sialyltransferase; TCA, trichloroacetyl; Tol, *p*‐tolyl; Tf, trifluoromethanesulfonyl.

Establishing structure–function relationships of glycans, or targeting specific glycans, requires access to sufficient amounts of well‐defined glycans. While a few glycans can be isolated in pure form from natural sources [63, 64], the heterogeneity of natural glycan structures usually prevents their separation. Unlike peptides or nucleic acids, glycans consist of differently linked monosaccharide units. The synthetic approaches toward glycans need to proceed regioselectively (i.e., forming exclusively (1→2), (1→3), (1→4) or (1→6) linkage) and stereoselectively, as any of the glycosidic bonds can be axial or equatorial. Some glycans even feature phosphodiester bonds [65, 66, 67, 68], that can be, in contrast to nucleic acids, linked axially or equatorially [69]. Glycans are often decorated with different modifications (i.e., phosphate [70], sulfate [71], esters [72] and others [73, 74, 75]) and these modifications have in numerous cases proven to be essential for their biological roles [71, 73, 76]. Due to the structural diversity of glycans, many ways evolved to overcome the synthetic challenges associated with the complex glycan structures (Figure 2).

### 2.2 Solution‐Phase Synthesis

The traditional approach to glycan synthesis employs a reaction of an activated electrophilic glycosyl donor with a nucleophilic glycosyl acceptor in solution. Complete regioselectivity of glycosylation is usually achieved by protecting all but the desired hydroxyl of the acceptor. Some strategies employing minimally protected donors and acceptors have been developed as well [77, 78]. Achieving complete stereoselectivity can be much more challenging. (1→2)‐*trans* glycosidic linkages are easiest to install as stereo‐selectivity is ensured by neighboring ester group participation [79]. Some (1→2)‐*cis* linkages can still be formed selectively by benefitting from remote group participation [79, 80]. The most difficult (1→2)‐*cis* linkages cannot take advantage of neighboring or remote group participation (e.g., β‐mannosylation and β‐L‐rhamnosylation) and have to rely on operationally more complex strategies [81]. These include Crich β‐mannosylation that proceeds through axial anomeric triflate [82], intramolecular aglycon delivery [83, 84], hydrogen‐bond‐mediated intramolecular aglycon delivery [85, 86], chiral auxiliaries or additive reactivity modulation, as well as many others [87].

Chemical installation of sialic acid (e.g., Neu5Ac or Neu5Gc) is often accompanied by low stereoselectivity due to the lack of *O*‐3 to support participating group. This complexity inspired synthetic chemists to develop various strategies [88] including enzymatic sialylation (see Section 2.5). Many complex glycans containing sialic acid were synthesized in solution (e.g., *N*‐glycans [89]). Solution synthesis has relied on a range of protecting groups for branching and late‐stage modifications, as was demonstrated by preparing a heparan sulfate library [71]. Starting from a tetrasaccharide with six orthogonal protecting groups, all 64 sulfation patterns were constructed to decode glycosaminoglycan–protein interactions.

Solution phase syntheses have been employed to construct glycans containing unusual monosaccharides [90, 91, 92, 93, 94], unusual [93] or unnatural modifications [95, 96], many modifications [97] or challenging linkages [91, 93, 98]. Solution syntheses allow for ready scale‐up [99] as they do not require complete stereoselectivity. On the downside, such solution phase syntheses are labor intensive as they require purification after each synthetic step.

### 2.3 One‐Pot Synthesis

Wong's group systematically studied the reactivity of thioglycoside donors and established relative reactivity values (RRV) [100, 101]. When two donors of sufficiently different reactivity are treated with activator, the more reactive (higher RRV) donor is activated to selectively glycosylate the less reactive BB. The approach was illustrated in the context of one‐pot synthesis of heparin pentasaccharide [102] or oligo(*N*‐acetyllactosamine) (LacNAc) [103]. A software for selecting BBs based on RRVs was developed to suggest one‐pot synthesis of oligosaccharides [104]. This approach simplified some syntheses [102], but often only two successive glycosylations can be reliably pipelined while putting constraints on BB design to differentiate the RRVs. Iterative activation of the donor prior to acceptor addition allows for several successive glycosylations in one‐pot and reduces the synthesis time and purification efforts. Activating systems were developed to render this approach independent of glycosyl donor reactivity [105]. Pre‐activation of thioglycosides enables five successive glycosylations in one‐pot fashion on gram scale [106]. Employing a convergent strategy, a branched 92‐mer mycobacterial arabinogalactan (i.e., polymer of arabinose (Ara) and galactose monosaccharides) was prepared. Later, a 140‐mer was assembled from galactopyranose and arabinofuranose BBs [107].

### 2.4 Automated Glycan Synthesis

A synthesizer was developed to execute the iterative pre‐activation strategy and synthesize an arabinofuranose 1080‐mer by multiplicative synthesis [108]. Other protected biologically relevant glycans including Globo‐H (α‐Fuc‐(1→2)‐β‐Gal‐(1→3)‐β‐GalNAc‐(1→3)‐α‐Gal‐(1→4)‐β‐Gal‐(1→4)‐β‐Glc) and fucosyl‐GM1 hexasaccharides or Lewis^X^ and Lewis^Y^ structures were prepared as well. While the pre‐activation methods are powerful for accessing complex glycans like protected fondaparinux (marketed synthetic anticoagulant) pentasaccharide on gram scale [108], many syntheses rely on di‐ or tri‐saccharide BBs [108]. Other automated solution phase approaches were developed using HPLC setup [109], fluorous tags [110, 111, 112] or an electrochemical cell [113, 114].

Solid‐phase synthesis lends itself particularly well to automation as excess building blocks can be used and reagents are simply washed away. Automated glycan assembly (AGA) uses synthesizers [115] to automate on‐resin glycan synthesis. The synthesis relies on iterative repetition of acidic wash to neutralize any base from previous steps, glycosylation, optional capping of the deletion sequence, and removal of the temporary protecting group [116]. The constructed glycan is cleaved from the solid support using UV light and deprotected in solution [117]. Using only two types of permanent protecting groups for BB design, ester (participating) and benzyl ether (non‐participating), enables a convenient two‐step total‐deprotection strategy with single purification of the final glycans [118, 119, 120]. The purified glycans can then be conjugated through a 5‐aminopentanol linker (e.g., to carrier protein) in solution.

AGA is a versatile tool to access complex glycans and is much simpler and faster than solution‐phase methods [116]. AGA enabled the construction of Lewis‐type antigens [121]. To illustrate the efficiency of the synthetic process, a linear α‐(1→6)‐mannan 100‐mer was prepared by AGA, while a 31‐mer of α‐(1→2)‐branched α‐(1→6)‐mannan demonstrated that even large structures can be readily constructed [122]. Most common hexopyranose [116] and several furanoside [123] BBs have been employed in AGA. Systematic BB development allowed for the automated construction of amylose featuring up to 20 *cis*‐glycosidic linkages [79]. Using modified BBs, glycans bearing fluorine labels [124], unnatural charged carboxylates and amines [125], or conformationally constrained by various staples [118, 120], were constructed by AGA. On‐resin glycan modification strategies provide streamlined access to glycans containing a mannose‐6‐phosphate unit [126] or up to 11 sulfate esters [119]. Rethinking the attachment of the glycan to the resin opened a direct way to reducing end‐functionalized glycans, including saponins and solid‐phase peptide synthesis (SPPS)‐compatible *O*‐glycans [127]. AGA offers streamlined access to glycan collections ideal for fast screening [116]. While judicious protecting group selection allows for efficient access to small collections of glycans by solution synthesis [71], synthesis of longer saccharides may largely profit from automation. Although the scale of AGA is limited [128], the synthesis of leads can be scaled‐up in solution, using the same BBs.

While most of human glycosidic linkages have been already implemented in AGA, synthesis of bacterial glycans still poses a challenge for automation, and only some bacterial glycans are accessed currently by AGA [116]. Progress is hampered by bacterial glycan diversity of in terms of rare monosaccharides, unusual modifications and challenging linkages (see Chapters 1 and 2.1) [54, 129].

### 2.5 Chemoenzymatic Synthesis

The efficient and selective chemical construction of certain linkages or specific modifications remains difficult [130]. Chemoenzymatic synthesis leverages the unique catalytic capabilities of enzymes to attach unprotected monosaccharides with high regio‐ and stereo‐selectivity. Typically, glycosyltransferases are used to transfer monosaccharide units from sugar nucleotides that can be generated in situ [131, 132]. Chemical synthesis is often used to prepare the core oligosaccharide that is, extended or modified enzymatically [133].

Enzymatic sialylation became a standard to overcome the difficulties associated with the chemical incorporation of sialic acid. Hybrid‐type *N*‐glycans, constructed by solution‐phase synthesis, were α‐(2→3)‐ and α‐(2→6)‐sialylated using the corresponding sialyltransferases [59]. A chemoenzymatic approach was used to construct a collection of ganglio‐oligosaccharides [134]. α‐(2→3,6,8,9)‐Neuraminidase A was used to remove internal Neu5Ac residue that was necessary for enzymatic installation of terminal galactose, illustrating the synthetic limitations arising from the substrate specificity of enzymes. A collection of sulfated gangliosides was accessed by three successive chemical glycosylations to install α‐(2→8)‐linked Neu5Ac trimer while the terminal sialic acids were installed enzymatically [135].

Enzymes can also be leveraged to incorporate chemically modified monosaccharides to introduce bio‐orthogonal chemical tags such as azides or alkynes [136, 137]. A chemoenzymatic synthesis of GD2 (α‐Neu5Ac‐(2→8)‐α‐Neu5Ac‐(2→3)‐[β‐GalNAc‐(1→4)]‐β‐Gal‐(1→4)‐Glc) pentasaccharide and its 9‐NHAc derivative, demonstrated that the C‐9 position modification is tolerated by α‐(2→8)‐sialyltransferase [138]. A series of azido‐Globo‐H analogs was prepared starting from lactose, by employing galactose oxidase to oxidize C‐6 to aldehyde followed by reductive amination and diazo‐transfer [131]. Introduction of 6‐azidofucose was well tolerated by the corresponding transferase. Using fluorinated GlcNAc, Gal and Fuc, a collection of fluorinated Lewis^x^ derivatives was prepared in one‐pot [132]. The chemoenzymatic approach was used to efficiently prepare natural glycans on a large scale. Mimicking the biosynthetic pathway of *N*‐glycan synthesis, a concise chemoenzymatic synthesis of symmetric and asymmetric biantennary *N*‐glycans was achieved [139].

Although enzymes display excellent substrate specificity and regioselectivity, more than one enzyme substrate may be present in a glycan chain, necessitating the use of protecting groups. In chemoenzymatic synthesis of *N*‐glycans, peracetylated GlcNAc [140], 4‐*O*‐Ac‐GlcNAc [141], GlcN and 2‐deoxy‐2‐azidogluocose (GlcN_3_) [142] were employed to mask the monosaccharide units during enzymatic extension. GlcN_3_ has been used to access complex asymmetric human milk oligosaccharides [143], Ketodeoxynonulonic acid (KDN) sialic acid, Neu5Ac, and oxidation to aldehyde protected galactose to selectively modify poly‐LacNAc [144]. Chemoenzymatic syntheses were automated using temperature‐dependent polymer and a modified peptide synthesizer [145]. An automated platform simplified enzymatic glycan syntheses using a sulfonate tag [146].

Although chemoenzymatic synthesis enables easier access to complex structures in comparison with other methods, a major challenge for all chemoenzymatic approaches is enzyme availability. While enzymes are highly specific, they are limited by substrate specificity and generally require longer reaction times.

In summary, recent advances in glycan synthesis have greatly expanded access to well‐defined, complex synthetic glycans, facilitating biomedical investigations. Below, we review the use of these glycans for the development of diagnostic and therapeutic tools.

## 3 Synthetic Glycan Microarrays

### 3.1 Recent Advances in Glycan Microarray Technology

Glycan microarrays consist of glycans from synthetic or native sources immobilized on a surface to screen for Ab binding in a high‐throughput and high‐content manner [147, 148]. Since the first reports of glycan microarrays [149, 150], arrays of various formats have been developed. Optimized glycan presentation, comprehensive high‐content, and high‐throughput analysis of samples in functional assays have been achieved. Among the most common formats are the printed microarrays, with synthetic glycans attached covalently‐ or non‐covalently onto surface‐modified glass slides [147, 149, 151, 152]. Less common array formats include glycodendrimers [153], glyco (deoxyribonucleic acid) DNA arrays [154, 155], glycoclusters [155], and glycopolymer arrays [156]. The latest advancements include multivalent and multiplex formats with the Luminex bead arrays [157, 158] and the liquid glycan array (LiGA), also suitable for studying glycan‐binding proteins (GBPs) both on cells and in vivo [159]. Glycan microarray slides can be modified in situ with various glycoenzymes to manipulate glycan structures and study their interactions with GBPs [160, 161]. Physicochemical treatments such as with sodium dodecyl sulfate (SDS) and microwaves have been explored to reuse glycan microarray slides [162]. The different glycan microarray formats have been extensively reviewed recently [163, 164]. In the following section, we will focus on characterization of anti‐glycan Abs by glycan microarrays for the discovery of biomarkers for diagnostic, prognostic, and therapeutic purposes.

### 3.2 Glycan Microarrays to Investigate Anti‐Glycan‐Ab Repertoires

Endogenous anti‐glycan Abs hold great potential for biomedical applications. However, biomarker discovery and vaccine development require proper controls and baseline level determination in healthy individuals. Notably, baseline levels of anti‐glycan Abs vary with age, gender, ethnicity, and blood type [148, 165], emphasizing the critical need for their in‐depth profiling in large patient cohorts. Large glycan microarrays with over 1000 structures have shown that the anti‐glycan Ab repertoire is unique to each individual, influenced by antigen exposure and Ab generation capacity. With age, IgM repertoires show increasing similarity [166]. Some of the most prominent anti‐glycan antibodies found in human serum of adults are directed against the immunogenic glycan epitopes αGal (α‐Gal‐(1→3)‐Gal), L‐rhamnose (Rha), and different blood group antigens [167].

Germline antibodies are generally poly‐reactive compared to their affinity‐matured counterparts [168, 169]. Using glycan microarrays to study endogenous germline Abs in mice, Delaitsch et al. revealed that most glycan‐binding Abs selectively recognized clinically relevant TACAs and blood group antigens rather than being poly‐reactive [170]. These studies provide critical insights into anti‐glycan Abs and highlight the diversity of anti‐carbohydrate immune responses and pave the way for the characterization of endogenous anti‐glycan Ab repertoires.

### 3.3 Anti‐Glycan Abs as Biomarkers

Biomarker identification enables the detection of disease, monitoring of disease progression, and the efficient assessment of treatment responsiveness and patient prognosis. Moreover, it can lead to novel criteria for patient stratification and personalized treatment tailored to the patient's unique characteristics and medical needs [171]. The use of well‐defined synthetic glycans immobilized on microarrays, beads, or phages forms high‐throughput tools for profiling and investigation of anti‐glycan Abs as biomarkers in body fluids such as blood, breast milk [92], cerebrospinal fluidx [172], saliva [173, 174], amniotic fluid [175] or cervicovaginal fluid [175, 176, 177]. Antibody profiling has been investigated in infectious diseases and cancer, highlighting their vast potential for disease identification and patient stratification.

#### 3.3.1 Anti‐Glycan Abs in Infectious Diseases

Anti‐glycan Abs in infectious diseases have been studied extensively. *Mycobacterium tuberculosis* (Mtb) infection can lead to active or latent tuberculosis. Glycan microarrays were used to differentiate Ab binding profiles of patients or animals developing these disease forms. Higher anti‐arabinomannan (AM) IgG levels targeting motifs with terminal arabinofuranose residues correlated with favorable outcomes. In asymptomatic patients, elevated anti‐AM IgG levels were linked to Ab‐mediated protection [178]. Ab responses in 57 cynomolgus macaques after low‐dose Mtb infection showed that animals with higher preexisting airway and plasma IgA against specific AM motifs developed latent rather than active TB [179].

Although opsonic IgG Abs targeting *Staphylococcus aureus (S. aureus)* showed efficacy in animals, they failed clinically [180]. WTA‐specific Abs were analyzed using multiplexed bead‐based assays with synthetic ribitol phosphate‐ WTA hexamers. WTA‐specific IgM levels correlated inversely with mortality and impaired opsonization, while IgM surpassed IgG in opsonophagocytic killing assays [181]. These findings emphasize the need to assess various Ab classes for patient risk stratification and further mAb design.

#### 3.3.2 Anti‐Glycan‐Abs as Cancer Biomarkers

Research on circulating anti‐glycan Abs as cancer biomarkers using glycan microarrays has been reviewed extensively [164]. The majority of Food and Drug Administration (FDA)‐approved biomarkers for cancer detection are based on carbohydrates or glycoproteins [171, 182, 183, 184, 185, 186, 187]. These biomarkers highlight the importance of glycosylation changes in cancer. Anti‐glycan Abs that reflect the aberrant glycosylation characteristic of malignancies highlight the potential as cancer biomarkers. The sera from ovarian cancer patients at different stages, benign tumor patients, and control donors were examined using a microarray containing defined *O*‐ and *N*‐linked glycans, glycolipids, and natural glycoproteins. A significant increase in IgG antibodies against 6‐*O*‐sulfated‐Lewis^c^ and the glycosphingolipid trisaccharide P_1_ (α‐Gal‐(1→4)‐β‐Gal‐(1→4)‐β‐GlcNAc), and in IgMs against Lewis^y^ in the diseased versus the control group were observed [188].

#### 3.3.3 Biomarkers to Predict and Monitor Autoimmunity and Immune Deficiencies

The immune system can generate Abs against self‐antigens, leading to various autoimmune diseases [189, 190]. In many cases, the targets and function of these Abs remain unknown, making glycan microarrays valuable for biomarker discovery. Interestingly, aberrant Ab glycosylation [191] and anti‐glycan Abs have been identified in several autoimmune conditions. In multiple sclerosis (MS), the mechanisms behind autoantibody formation remain unclear. Sera from 25 relapsing–remitting MS (RRMS) patients and controls were screened on glycan microarrays, revealing elevated IgG reactivity to dietary Neu5Gc and self‐glycan Neu5Ac in RRMS patients [192]. Anti‐carbohydrate Abs have been linked to the progression of type 1 diabetes [193]. Clusters enriched for aminoglycosides, blood group A/B antigens, glycolipids, ganglio‐series, and O‐linked glycans correlated with disease progression and are potential biomarkers [193]. In Huntington's disease (HD), ganglioside‐focused glycan microarrays showed higher anti‐GD1b autoantibody levels in pre‐manifest HD patients compared to controls and symptomatic individuals [194]. Primary antibody deficiencies impair humoral responses to carbohydrate antigens, causing infections, dysbiosis, and autoimmunity. Glycan microarrays of serum samples revealed loss of anti‐ α‐Gal‐ and GalNAc‐ terminated Abs reactivity and disease‐specific reactivity against microbial antigens, self‐antigens, and TACAs [195]. Rare autoimmune disorders such as autoimmune cytopenia [196], anti‐MAG neuropathy [197], and Duchenne muscular dystrophy [198] also involve aberrant anti‐glycan‐antibody levels.

### 3.4 Development of Novel Diagnostic Tools Based on Anti‐Glycan Abs

Biomarker screening studies using glycan microarrays formed the basis for the development of novel tools in various formats to detect anti‐glycan Abs as novel biomarkers in body fluids of human and animals. A rapid test to detect brucellosis, a zoonotic disease caused by various *Brucella* species, in bovine serum was developed. The test, based on a fluorescence polarization assay containing a synthetic fluorescein‐labeled trisaccharide tracer, achieved similar diagnostic efficiencies as traditional, time consuming, methods [199]. A bead‐based multiplex assay using synthetic replicates of GPI glycolipids that cover the surface of *Toxoplasma gondii* parasites covered to beads was employed to measure IgGs and IgMs in patient sera. Significant anti‐GPI IgM titers during the early acute parasitic infection can be used to detect infection [200]. Leprosy, a chronic bacterial infectious disease caused by *Mycobacterium leprae* or *M. lepromatosis* can be diagnosed using synthetic phenolic glycolipids to detect IgM in patient sera by enzyme‐linked immunosorbent assay (ELISA) and a synthetic‐glycan based lateral flow assay [201].

A waveguide‐mode sensor was used for the detection of serum Abs against type A and type B oligosaccharide antigens on the surface of red blood cells. Immobilizing a synthetic trisaccharide blood antigen on a sensor chip helped to observe significant changes in the reflectance spectra in the presence of serum anti‐A/B antigen Abs. This technology could be included in a portable device for rapid blood testing [202]. Synthetic HNK‐1, a trisaccharide carbohydrate epitope, is included in the BÜHLMANN GanglioCombi MAG ELISA kit, used to detect anti‐MAG IgMs for the diagnosis of autoimmune demyelinating neuropathies [197, 203].

## 4 Active Immunization

Glycoconjugate vaccines, and immunization with native or synthetic glycans conjugated to synthetic or protein carrier, have demonstrated tremendous potential in preventing bacterial diseases and treating cancer. The vaccines initiate the production of protective Abs by inducing a T cell‐dependent immune response and the formation of memory B cells. Further enhancement of the immune response is achieved by adding adjuvants that boost the immune response in a non‐specific way. Commercial conjugate vaccines containing native CPS are successful in preventing infections caused by *Streptococcus pneumoniae (S. pneumoniae)* [204]*, Haemophilus influenzae* type b (Hib) [45], *Salmonella typh*i [205], and *Neisseria meningitidis (N. meningitidis)* [206].

With the rising global mortality due to bacterial and fungal infections and associated drug resistance, novel treatment approaches are in high demand [60, 61]. Poor immunogenicity and serotype replacement threaten the efficacy of existing vaccines [204].

With cancer incidence and mortality increasing [62], vaccines against carbohydrate antigens on cancerous cells are a promising means for targeted therapy in vivo [50]. TACAs exhibit structural similarities to glycans on healthy cells, and thus, are often tolerated by the immune system, posing significant challenges in the design of TACA‐based vaccines [32, 207]. Cancer vaccines should overcome immune tolerance while inducing a robust T‐cell response that selectively targets tumor cells without damaging healthy tissues.

Approaches based on well‐defined synthetic glycans can substantially help overcome some of the challenges of vaccine design. Due to their defined structure and homogeneity, synthetic glycans represent safer and more effective vaccine candidates. Synthetic and semisynthetic vaccine candidates help to determine the minimal required length, frameshift, and necessary modifications of the glycan backbone to induce the formation of functional and protective antibodies [208]. Recent advances in semi‐synthetic microbial [54, 208] and cancer [209] carbohydrate‐based vaccine candidates have been thoroughly reviewed recently.

**FIGURE 3:**
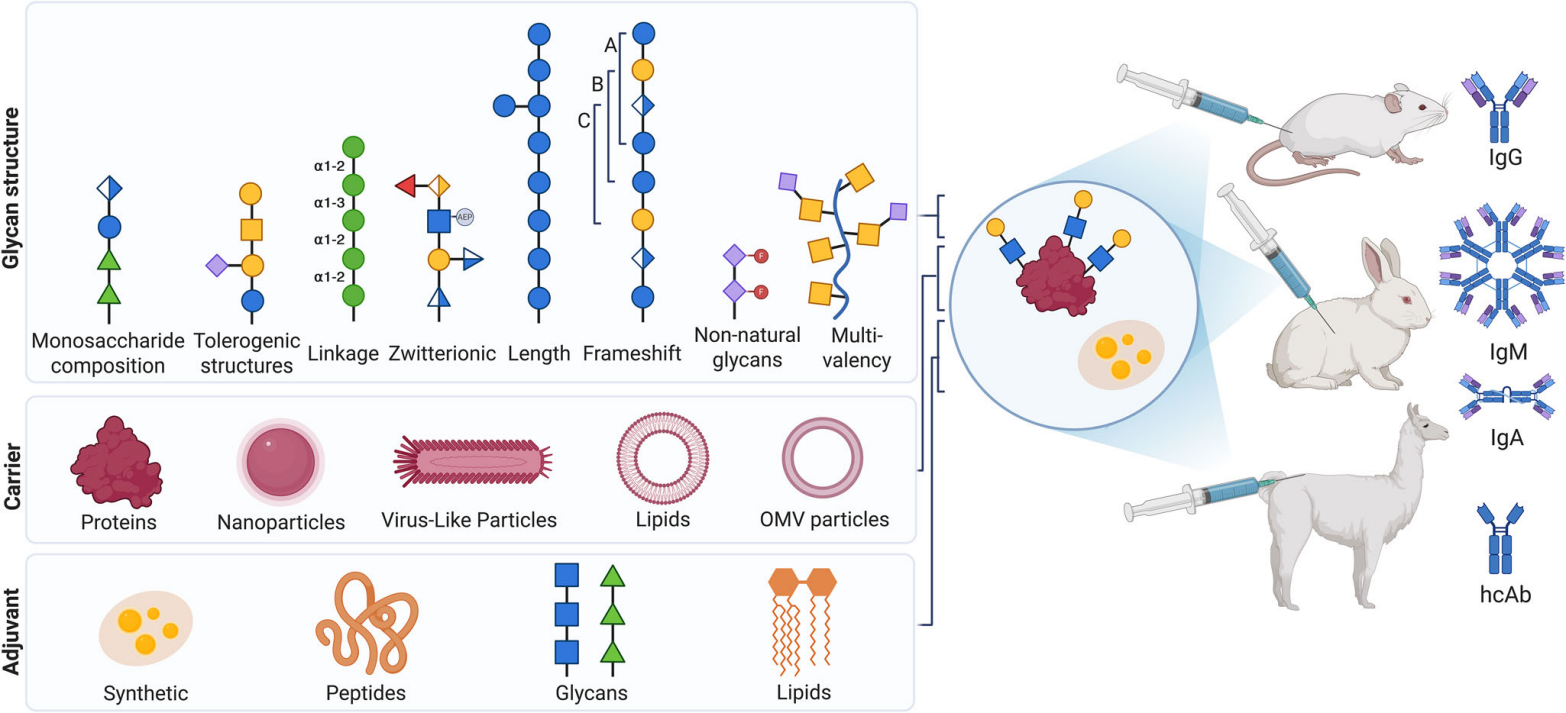
Essential considerations for effective vaccine design to generate monoclonal anti‐glycan Abs. OMV, outer membrane vesicle; hcAb, heavy‐chain only antibody.

The basis for mAb development is often the immunization of an animal or human with a vaccine candidate or novel epitope. Several considerations are important for vaccine design and the development of functional mAbs. Here, we focus on considerations and advances for the generation of synthetic and semisynthetic carbohydrate vaccines to fight infectious pathogens and cancer (Figure 3).

### 4.1 Rational Design of Synthetic Glycan Vaccines for Optimal Antibody Generation

#### 4.1.1 Structure

The immunogenicity of glycoconjugates is defined by the monosaccharide composition, linkages, and branches, that are often specific for the organism [11]. These glycans can elicit a distinct immune response depending on the immunological context, differing according to whether they are recognized as self or foreign to the host and their interaction with the immune system. Foreign microbial structures such as rhamnose are readily recognized by the human immune system and induce antibody formation. Other structures generated with the 10 human monosaccharides, even if aberrant as is the case for TACAs, are less immunogenic in humans. The sialic acid Neu5Ac is even tolerogenic, involved in impaired immune surveillance [12]. Additional diversity is added by glycan linkages that define the glycan conformation and lead to a vast diversity of potential glycan epitopes. Bacterial and fungal pathogens express unique enzymes that generate glycosidic linkages not found in humans, such as the β‐(1→3) or β‐(1→6) linkages between glucose residues, or the complex α‐(1→6) and β‐(1→2)‐linked mannose repeats, forming cell wall β‐glucans and mannans, respectively [11, 210]. *Candida*‐infected patients produce different IgG levels directed against structurally distinct synthetic β‐glucans and mannans, indicating differing immunogenicity [211]. Certain bacteria contain zwitterionic glycans, a special type of glycans with both positive and negative charges within repeating units, able to elicit MHC‐II and T cell‐dependent responses [212]. Zwitterionic structures have been synthesized and hold potential in synthetic, fully‐carbohydrate vaccines [213].

##### 4.1.1.1 Length and Identifying Minimal Epitopes

The glycans coating the bacterial and fungal cell walls, are composed of several repeating units. The immunogenicity of an oligosaccharide often depends on the length of the glycan epitope and the minimal epitope required for mAb binding [208]. Well‐defined synthetic glycans enable the experimental determination of minimal epitopes and optimal glycan length.

A tetrasaccharide unit is the minimal epitope to target *Pseudomonas aeruginosa* and is able to elicit high Ab titers in mice [214]. The→3)‐β‐D‐ManNAcA(4‐OAc‐1→3)‐α‐L‐FucNAc‐(1→3)‐α‐D‐FucNAc‐(1→repeating unit of *S. aureus* CPS 8 and three repeating units are essential to generate an adequate immune response [215]. In case of *Candida auris* a β‐Man‐(1→2)‐Man disaccharide was identified as a lead for vaccine development [69].

A semisynthetic vaccine candidate related to the *Klebsiella pneumoniae (K. pneumoniae)* O‐antigen O2afg was based on the primary O‐antigen type found in carbapenem‐resistant *K. pneumoniae* ST258. Functional IgG Abs were induced in rabbits after immunization with a glycoconjugate containing two repeating units. These mAbs protected mice from acute pneumonia in vivo [216]. Semisynthetic polysaccharide vaccine candidates against the CPS of *Haemophilus influenzae* serotype a (Hia) containing up to five phosphodiester‐linked→4)‐β‐D‐Glc‐(1→4)‐D‐ribitol‐5‐(PO_4_→ repeating units were generated. All glycoconjugates induced Hia targeting Abs in rats, without showing an explicit length dependency [66].

##### 4.1.1.2 Frameshift

Changing the frameshift of the repeating unit and investigating the terminal glycan to optimize the efficacy of the synthetic vaccine and immune response is crucial. Glycans at the nonreducing terminus make up only a small part of the overall glycan structure and are believed to be most important for immunogenicity. Such glycans determine the recognition pattern from antibodies depending on the glycan frame (e.g., in generic terms: ABC, BCA, or CAB). The altered glycan composition or frameshift affects the glycan epitope and the effectiveness of inducing a protective immune response.

*Escherichia coli* (*E. coli)* O25B is a major serotype responsible for extraintestinal infection with *E. coli*. Recently, Naini and colleagues synthesized five oligosaccharide antigens of different lengths and with two different frameshifts. The resulting semisynthetic glycoconjugates induced functional IgG Abs in mice and demonstrated opsonophagocytic activity in vitro. One frameshift was superior and induced higher IgG titers with stronger opsonophagocytic activity and a higher affinity [57]. Different glycan frameshifts of *S. pneumoniae* ST8 have been synthesized, and serum antibody binding was evaluated via glycan microarray. The superior tetrasaccharide frameshift was selected to generate an ST8 vaccine that induced protective antibodies in mice and in rabbits [217].

##### 4.1.1.3 Glycan Modifications

Synthetic glycans provide a unique opportunity to conduct in‐depth studies on how chemical groups and artificial analogues of native structures can enhance vaccine design and efficacy. Bacterial glycans often contain acetates, pyruvates, or other modifications [208]. Mammalian structures may contain post‐glycosylation modifications such as sulfation, acetylation, methylation, and phosphorylation [218]. Glycan modifications impact immunogenicity, immune tolerance, and stability.

Acetylation of glycan subunits was important for mAb binding of *Acinetobacter baumannii* 17978 [72], while it did not affect mAb binding in an *E. coli* STO25B vaccine candidate [57]. Acetamido derivatives were investigated on STO5 O‐antigen of *P. aeruginosa* [93] and STO51 O‐antigen of *Plesiomonas shigelloides* [219]. Synthetic trisaccharide repeating units were key antigenic epitopes, while the acetamidino group did not influence the antigenicity and Ab binding. In a similar O‐antigen study, a tetrasaccharide related to the *Shigella dysenteriae* ST10 O‐antigen showed that the (*S*)‐4,6‐*O*‐pyruvyl ketal is an essential structural feature [75]. Poly(β‐(1→6)‐*N*‐acetylglucosamine) (PNAG) is a structurally heterogeneous polymer expressed on many pathogens. A set of 32 PNAG pentasaccharides was employed to study the impact of the location of free amine groups and *N*‐acetylation on antigenicity. Two lead structures helped to develop a vaccine candidate against methicillin‐resistant S. *aureus*, that showed protection in an in vivo infection model [220].

Glycan derivatives of TACAs have been generated and used in vaccine development to induce an immune response restricted to tumors and reduce side effects. For instance, acetylation of GD2 and GD3 (α‐Neu5Ac‐(2→8)‐α‐Neu5Ac‐(2→3)‐β‐Gal‐(1→4)‐Glc) was used to generate NHAc‐GD2 and NHAc‐GD3 glycoconjugate vaccines, which elicited strong, long‐lasting IgG Ab response with few side effects [138, 221].

Additionally, entirely artificial groups have been explored in microbial and tumor vaccine development. Advantages include enhanced antigenicity, regulation of glycosylation, increased enzymatic stability, and facilitating structural determination [222]. Fluorination of sialic acid in a disaccharide improved the efficacy of a vaccine candidate against *N. meningitidis* serogroups B (MenB) and/or C (MenC). Endogenous anti‐glycan Abs against the non‐native fluorinated synthetic glycans were formed in an in vivo mouse model, and were protective in vitro against MenB and/or MenC [95]. Other groups used this strategy to generate vaccines of derivatives of the TACA Globo‐H. Glycoconjugate vaccines with different fluorine‐modified *N*‐acyl Globo‐H analogues showed that di‐ and tri‐ fluorine‐modified *N*‐acyl Globo H conjugates induced higher IgG levels than the monofluorinated or the unmodified Globo‐H glycoconjugate [223]. Evaluation of several azido‐Globo‐H glycoconjugates showed that the glycoconjugate with azide at Gal‐C6 of lactose induced the strongest immune response [131].

#### 4.1.2 Carrier

As short glycan structures alone are T cell independent antigens, glycans need to be conjugated to a carrier to enhance the immune response. Most glycoconjugate vaccines utilize the FDA‐approved carrier protein cross‐reactive material 197 (CRM197), diphtheria toxoid (DT), or tetanus toxoid (TT). Their main obstacle is carrier‐induced epitopic suppression. The peptide or carrier parts of the vaccine candidate compete for the carrier‐specific primed helper cells, resulting in an increase in the Ab response to the carrier, while the immune response to the polysaccharide is decreased resulting in impaired immune responses to vaccines conjugated with the same carrier [224, 225]. Current efforts focus on the development of cost‐effective engineered or synthetic carriers with simplified manufacturing and high activity [54].

##### 4.1.2.1 Glycoproteins

Replacing the carrier protein with a pathogen‐specific protein can enhance the immune response and provide additional protection across different serotypes. This concept was explored for a vaccine candidate against *S. pneumoniae* as synthetic glycans were conjugated to two serotype‐independent conserved antigens: pneumolysin and pneumococcal surface protein A. The conjugates were able to reduce disease severity in a mouse challenge model [226]. Similarly, porin A, a major outer membrane protein of *N. meningitidis* was explored as a pathogen‐specific carrier protein against *N. meningitidis*. The conjugates demonstrated stable, long‐lasting, and protective IgG response in mice [95].

In cancer, small TACAs, such as Tn, sTn, TF, and sTF, pose significant challenges for vaccine design. These minimal structures are poorly immunogenic, may be subject to immune tolerance, and fail to replicate their natural presentation on tumor cells. To replicate the natural, multivalent displays, cancer‐relevant carbohydrates embedded in their natural peptide backbones like mucins (e.g., MUC‐1, MUC‐4) or CD44 were synthesized. A modular tripartite conjugate composed of a GalNAc glycocluster on a tetraphenylethylene scaffold, and a Tn‐decorated MUC‐1 glycopeptide [MUC‐1(Tn)_3_], conjugated to a TT‐derived peptide were prepared. In vivo evaluation of the glycoconjugate revealed high IgG titers, binding strongly to various MUC‐1 structures on microarrays and MUC‐1 positive breast cancer cells [227]. The development of synthetic tumor‐associated mucin glycopeptide vaccines has been reviewed [228]. Enzymatically synthesized glycopeptides of CD44 carrying Tn and sTn *O*‐glycans, conjugated to different carriers were immunologically evaluated in mice. The multivalent conjugate elicited IgG Abs with reactivity to the synthetic CD44s‐Tn glycopeptides, CD44s‐Tn glycoengineered cells, and human tumors [229].

##### 4.1.2.2 Nanoparticles

Various nanoparticles offer multivalent epitope display and efficient adsorption by antigen presenting cells [230]. A TF (β‐Gal‐(1→3)‐GalNAc) ‐glycosylated MUC‐4 gold nanoparticle conjugate elicited the production of antigen‐specific IgGs, T cell activation, and generation of anti‐tumor cytokines in mice [231]. Virus‐like particles (VLPs) have been tested as vaccine carriers. VLPs lack viral genetic material and are non‐infectious, enabling the multivalent display of glycan antigens and the generation of lower levels of anti‐carrier Abs, limiting the effect of carrier‐induced suppression [232]. Common VLPs include the bacteriophages M13, *Myxococcus xanthus* (MX)1, cowpea mosaic virus (CPMV) and Qβ. A Qβ mutant (mQβ), lacking some B cell epitopes to further reduce immune responses against the carrier was used for a PNAG‐based conjugate against *S. aureus* that showed superior immunogenicity compared to a TT glycoconjugate [220, 232]. A Tn cancer vaccine candidate showed lower anti‐carrier Abs than the Qβ WT or other widely used carriers such as keyhole limpet hemocyanin (KLH), CRM197, or TT [220, 232]. Notably, the Tn mQβ glycoconjugate resulted in an increased survival rate of 80% in a breast cancer mouse model compared to a 20% survival rate with the WT carrier [232]. The platform proved flexible with additional mQβ glycoconjugated TACAs [138, 232].

##### 4.1.2.3 Glycolipids

Liposomal carriers can be used to incorporate clusters of carbohydrate antigens on their surface and hence stimulate B cells. Different types of lipid carriers are based on the characteristics of the lipid, including liposomes, lipid nanoparticles, glycolipids, and lipopeptides [208, 233]. The carriers have self‐adjuvating properties since they activate immune pathways involving toll‐like receptors and natural killer T cells. Such immunostimulatory lipids include 3‐*O*‐desacyl‐4ʹ‐ monophosphoryl lipid A (MPLA), the saponin QS‐21 and α‐galactosylceramide (α‐GalCer) as well as their analogues [230, 234]. Other liposomal carriers include S‐[(R)‐2,3‐dipalmitoyloxy‐propyl]‐*N*‐palmitoyl‐(R)‐cysteine (Pam_3_Cys), based on the immunologically active N‐terminal sequence of the principal lipoprotein of *E. coli* that has been used to develop a Tn vaccine candidate [235].

##### 4.1.2.4 Outer Membrane Vesicle‐Derived Particles

Outer membrane vesicles (OMVs) are naturally released nanoscale vesicles from Gram‐negative bacteria and an emerging vaccine carrier platform, combining intrinsic adjuvant activity with multivalent antigen display. Generalized modules for membrane antigens (GMMAs) are an engineered adaptation of OMVs, offering a low‐cost, genetically tunable, and scalable production of OMVs, with great potential for vaccine design [236]. The genetically modified bacteria can secrete GMMA‐OMVs that offer the possibility to carry, display, or be covalently coupled to a range of native or synthetic molecules such as LPS, peptidoglycan, viral RNA, or mammalian glycans [237, 238, 239, 240]. Their proven efficacy in animal models has led to several clinical trials testing the safety and immunogenicity of GMMA‐OMV vaccines against *Salmonella* and *Shigella* [241, 242]. Recently, Pesce et al. demonstrated that GMMA vesicles, coupled with synthetic MUC‐1 Tn/sTn mimetics, had a superior immunogenicity compared with CRM197‐conjugated Tn, and effectively protected mice against triple‐negative breast cancer [240]. Other synthetic‐glycan‐based GMMA‐OMVs platforms may broaden potential applications and pave the way for further developments, positioning this technology alongside nanoparticles and VLPs.

#### 4.1.3 Adjuvants

The choice of adjuvants is crucial for the development of semi‐synthetic and synthetic glycoconjugate vaccines. Natural and synthetic adjuvants, either as integral components or separate entities of the vaccine construct, enhance the immunogenicity of carbohydrate vaccines by triggering and modulating both innate and adaptive immune responses in an antigen‐specific manner [243]. Immunostimulatory adjuvants should have minimal toxicity, a bottleneck resulting in few adjuvants approved for clinical use. Aluminum salts (aluminum phosphate/hydroxide) are currently the only FDA‐approved adjuvants for human carbohydrate‐conjugate vaccines [244].

Different adjuvants and self‐adjuvating structures were explored for the development of vaccine leads against *N. meningitidis*. The impact of Freund's adjuvants and Alum on a fluorinated disaccharide was evaluated. Both adjuvants successfully activated the immune system in a T cell‐dependent manner [95]. A fully synthetic vaccine candidate against MenC combined α‐(2→9)‐linked di‐, tri‐, tetra‐, and pentasialic acids with MPLA. The constructs elicited a strong T cell dependent immune response in vivo comparable to that of other carbohydrate conjugate vaccine candidates [245].

Various adjuvant combinations were recently evaluated in a semisynthetic Globo‐H glycoconjugate. The adjuvants used included the saponin QS‐21, the α‐GalCer derivative S34, and their synthetic 3D‐MPLA. 3D‐MPLA and QS‐21 elicited a more potent immune response [246]. A novel class of adjuvants by conjugating α and β Rha to MPLA at 1’ and 6’ positions. Rha is capable of recruiting endogenous anti‐Rha Abs that are highly abundant in human sera. Different adjuvants potentiated antigen‐specific Ab production in mice. The covalently linked Rha and MPLA conjugates had a synergistic effect, and the α‐Rha linked to MPLA at 6’ was the best construct [247].

Built‐in adjuvants can improve immunogenicity. Fully synthetic Tn‐ Bovine serum albumin (BSA) conjugated to well‐defined chitotriose (CTS, β‐GlcNAc‐(1→4)‐β‐GlcNAc‐(1→4)‐GlcNAc) as a built‐in adjuvant (Tn‐BSA‐CTS) was superior to the Tn‐BSA vaccine co‐administered with CTS. Similarly, unimolecular Tn‐MUC‐1 (GVTSAPD(α‐GalNAc)TRPAPGSTA) with covalently linked synthetic saponin elicited a strong immune response and antigen‐specific IgGs [243]. Yeast‐derived β‐(1→3)‐glucans have successfully served as built‐in adjuvants in glycoconjugate vaccines [231]. Synthetic glycans, independent, and as built‐in adjuvants, are powerful tools to enhance the efficacy and improve the design of anticancer vaccines.

### 4.2 Recent and Ongoing Clinical Trials

Glycoconjugate vaccine development is a long, complex, and costly process [248]. Despite the many advantages of semisynthetic glycoconjugate vaccines, the only commercially available vaccine candidate targets Hib [249], while numerous candidates are undergoing clinical evaluation (Table 2).

**TABLE 2:** Synthetic and semi‐synthetic glycan vaccines in clinical development.

Vaccine Name	Target	Composition	Manufacturer/Responsible party of the clinical trial	Clinical trial	Phase
Quimi‐Hib	*Haemophilus influenzae* serotype b	polyribosylribitol phosphate (PRP)‐TT with aluminum phosphate adjuvant	Center for Genetic Engineering and Biotechnology (Cuba)	Marketed in Angola, Argentina, Brazil, Columbia, Cuba, Vietnam, India, Syria, Uruguay, Venezuela.	—
SF2a (GlycoShig3)	*Shigella flexneri* 2a	SF2a‐TT15 with/without Alhydrogel adjuvant	Institut Pasteur	NCT02797236, NCT04602975	II
IDOR‐1134‐2831	*Clostridium difficile*	—	Idorsia Pharmaceutical	2023‐506407‐26‐00	I
IDOR‐1142‐0810	*Klebsiella pneumoniae*	—	Idorsia Pharmaceutical	—	Preclinical
AV0328	PNAG oligoglucosamines	PNAG	Alopexx Vaccine	NCT02853617	I
OBI‐822 (Adagloxad Simolenin)	Globo‐H	Globo‐H‐KLH with OBI‐821 adjuvant	OBI Pharma	NCT01516307, NCT03562637	II, III
OBI‐833	Globo‐H	Globo‐H‐CRM197 with OBI‐821 adjuvant	OBI Pharma	NCT02310464 NCT05442060,	I, II
OBI‐833	Globo‐H	Globo‐H‐CRM197 with OBI‐821 adjuvant	OBI Pharma, Chang Gung Memorial Hospital	NCT06490198	II
OBI‐833	Globo‐H	Globo‐H‐CRM197 with OBI‐821 adjuvant	OBI Pharma, National Taiwan University Hospital	NCT05376423	II
Heptavalent conjugate vaccine	GM2, Globo‐H, Lewis^y^, TF, Tn, sTn, and glycosylated MUC‐1	Globo‐H‐GM2‐Lewis^y^‐MUC‐1‐sTn‐TF‐Tn‐KLH plus QS21 adjuvant	MSKCC	NCT00030823	Pilot trial
Unimolecular Pentavalent (Globo‐H‐GM2‐sTn‐TF‐Tn)	Globo‐H, GM2, sTn, TF, and Tn	Globo‐H‐GM2‐sTn‐TF‐Tn‐KLH plus QS‐21 adjuvant	MSKCC	NCT01248273	I
polyvalent‐KLH conjugate vaccine	GM2, Globo‐H, MUC‐1‐ Tn, TF	GM2‐KLH, Globo‐H‐KLH, Tn‐MUC‐1‐32mer‐KLH, TF‐KLH plus OPT‐821 adjuvant	MSKCC, Gynecologic Oncology Group	NCT00857545	II
polyvalent‐KLH conjugate vaccine	GM2, Globo‐H, MUC‐1‐ Tn, TF	GM2‐KLH, Globo‐H‐KLH, Tn‐MUC‐1‐32mer‐KLH, TF‐KLH plus OPT‐821 adjuvant	MSKCC	NCT01223235	Pilot
MAGTRIVACSEIN	Tn	Multiple antigenic glycopeptide displaying a Tri Tn glycotop plus AS15 adjuvant	Institut Pasteur	NCT02364492	I

Shigellosis is one of the most prevalent diarrheal diseases in low‐ and middle‐income countries. A vaccine candidate targeting *Shigella flexneri* 2a (SF2a) is composed of a synthetic 15mer oligosaccharide corresponding to three non‐*O*‐acetylated repeats linked to TT by a thiol‐maleimide spacer [99]. Phase I clinical data (NCT02797236) showed that the glycoconjugate vaccine candidate was safe and well tolerated in control individuals and induced functional Abs [250]. The vaccination induced long‐lived and class‐switched memory B cells of the IgG‐type [251, 252]. The vaccine candidate recently completed a Phase II clinical trial (NCT04602975). A Phase I clinical trial by Idorsia Pharmaceutical (2023‐506407‐26‐00) investigating synthetic glycan conjugates related to *Clostridium difficile (C. difficile)* (IDOR‐1134‐2831) has successfully been completed. A semi‐synthetic glycan vaccine candidate against *K. pneumoniae* (IDOR‐1142‐0810), is in preclinical development. A synthetic, broad spectrum‐vaccine candidate based on synthetic PNAG oligoglucosamines, has successfully completed a Phase I clinical trial (NCT02853617) sponsored by Alopexx Vaccine.

No anti‐cancer glycoconjugate vaccine has been approved to date. Nevertheless, advances in vaccine design and glycan synthesis have yielded promising candidates for cancer therapy. OBI Pharma developed Adagloxad Simolenin (OBI‐822), a synthetic Globo‐H–KLH conjugate vaccine. In a Phase II trial (NCT01516307) for metastatic breast cancer, the primary endpoints were not met; however, patients who developed strong anti‐Globo‐H Abs showed significantly improved survival [253]. Based on these findings, the company initiated a Phase III trial (NCT03562637) in early‐stage Globo‐H‐positive triple‐negative breast cancer, expected to be completed in 2027 [50]. OBI Pharma also developed OBI‐833, a Globo‐H–CRM197 conjugate, that completed a Phase I trial (NCT02310464) for gastric, lung, colorectal, and breast cancers, demonstrating good safety and immunogenicity [254, 255]. These results led to three Phase II trials: maintenance therapy for Globo‐H‐positive biliary tract cancer (NCT06490198), treatment of Globo‐H‐positive lung cancer with epidermal growth factor receptor (EGFR) mutation (NCT05442060), and treatment of Globo‐H‐positive esophageal cancer (NCT05376423). Several carbohydrate‐based cancer vaccines were explored at Memorial Sloan Kettering Cancer Center (MSKCC). A semi‐synthetic heptavalent KLH‐conjugate vaccine containing GM2, Globo‐H, Lewis^y^, TF, Tn, sTn, and glycosylated MUC‐1 proved safe and immunogenic in mice and humans in a pilot trial (NCT00030823) for ovarian, fallopian tube, or peritoneal cancers [256, 257]. Subsequently, a fully synthetic pentavalent conjugate targeting Globo‐H, GM2, sTn, TF, and Tn showed good immunogenicity and safety in a Phase I trial (NCT01248273) [258, 259]. A semi‐synthetic tetravalent version (Globo‐H, GM2, TF, MUC‐1‐Tn) reached Phase II trials (NCT00857545) but failed to improve survival [260], and a follow‐up pilot study (NCT01223235) showed good tolerance but no survival benefit [261]. A synthetic tetrameric glycopeptide displaying a tri Tn glycotope linked to a TT‐derived peptide (MAGTRIVACSEIN) completed a Phase I (NCT02364492) for localized breast cancer with high relapse risk, but no further developments have been reported [47, 262, 263, 264]. Despite challenges, synthetic TACA vaccines remain a transformative frontier in immunotherapy, driving continued progress in design and efficacy.

## 5 Passive Immunization

Natural passive immunization with endogenous Abs through placental transfer and in maternal breast milk is the first line of defense against pathogens from the early stages of human development [265]. In a medical context, Abs from external sources can be transferred to an individual to offer short‐lived and immediate protection against pathogens.

With the rise of antimicrobial resistance and cancer, passive immunization with narrow or broad‐spectrum Abs is becoming a valuable treatment alternative [266]. To date, passive immunization with poly‐ or monoclonal Abs is used to treat a range of human diseases, including infections, cancer, and autoimmune diseases. Moreover, Abs are administered as a preventive measure when the risk of infection or disease outbreak is high, especially to patients who are unable to independently generate protective Abs. mAbs are not only extensively employed for therapy but also extensively used in diagnostics and biomedical research.

### 5.1 Polyclonal Abs

Polyclonal Abs are used to treat infections, with the advantage of having different epitopes or Ab functionality to offer broad protection. Usually, polyclonal Abs are isolated from animals or humans that have been immunized with an epitope or have overcome an infection. Abs from animal sources can induce adverse effects and are not the preferred option for Ab therapies but rather are the basis for mAb development. Polyclonal Abs isolated from rabbits, immunized with a semisynthetic *K. pneumoniae* O2afg construct containing two repeating units of the →3)‐β‐D‐Gal*f*‐(1→3)‐[α‐D‐Gal*p*‐(1→4)]‐α‐D‐Gal*p*‐(1→ epitope, protected mice in a *K. pneumoniae* pneumonia model [216]. Polyclonal Abs targeting mammalian antigens have been produced. Polyclonal Abs against synthetic hypoglycosylated α‐dystroglycan mucin glycopeptides (NHAc‐GPTV(αMan)TIRG‐BSA) were explored for the diagnosis of dystroglycanopathies, a progressive degenerative muscular disease lacking accurate diagnostic tools. The Abs bound selectively to the synthetic glycopeptide in ELISA [267].

### 5.2 MAb Development With Synthetic Glycans

MAbs, derived from monoclonal cell lines, recognize specific antigens. Protein‐targeting Abs are successfully applied in preclinical and clinical settings [268]. In contrast, relatively few defined anti‐glycan Abs have been produced, often with lower affinity compared to peptide targeting antibodies, limiting their progression into clinical trials [269]. Recently, anti‐glycan antibodies with affinities in the nanomolar range have been produced [57, 58, 270].

**FIGURE 4:**
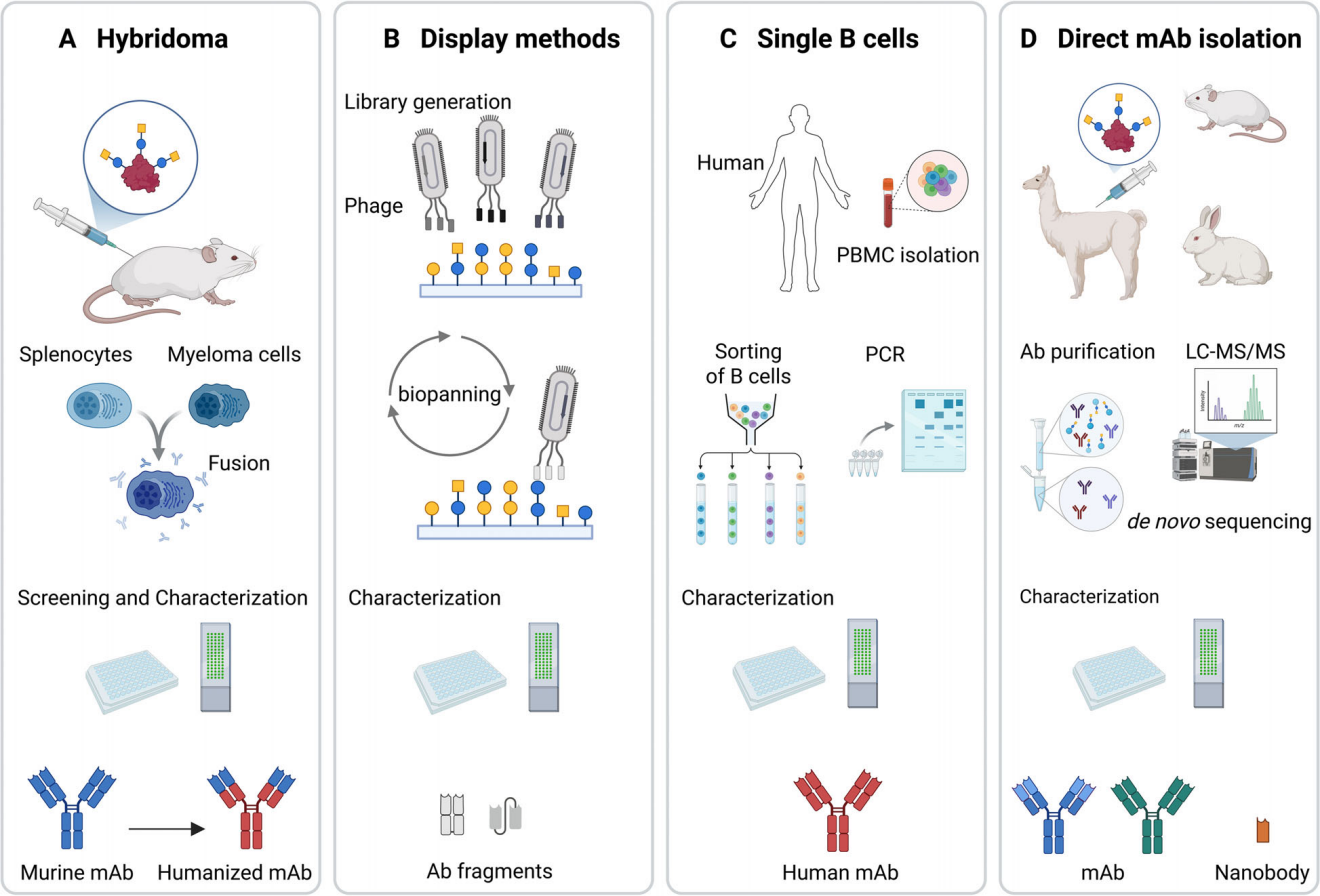
Prominent approaches for the generation and isolation of monoclonal anti‐glycan‐Abs. PBMC, Peripheral blood mononuclear cell; PCR, polymerase chain reaction, LC‐MS/MS: liquid chromatography–mass spectrometry.

Synthetic glycans enable the generation of mAbs against specific epitopes and help define the epitopes of glycan‐specific Abs developed using native glycans. Distinct methods enable the development of different antibody formats (Figure 4). The traditional approach entails immunizing an animal with the target epitope followed by the development of mAbs using the hybridoma technology (Figure 4A). B cells, harvested from the spleen, are then fused with immortal myeloma cells, a type of cancerous plasma cells. Cells that produce the desired Abs are selected and cloned to enhance mAb production [271]. Murine precursors must be humanized for use in human clinical trials, in a laborious, time‐consuming, and low‐throughput process. Humanization involves replacing animal‐derived sequences with human equivalents in order to reduce immunogenicity and toxicity for the patient [272]. This approach has been successfully used in the past to generate mAbs to target cancer [273] and infectious diseases [274].

Phage display is another method that uses the interaction between proteins presented on the surface of bacteriophages and other epitopes (Figure 4B) [275]. The proteome and genome are directly linked in bacteriophages, and the genome can be genetically modified so that the phages express Ab fragments on their surface. High‐affinity binders can then be determined by screening libraries of phages against the target antigen. The genetic material of the selected antigen‐binding phages is then isolated, sequenced, and larger amounts of the Ab fragments are produced. It is a high‐throughput and cost‐effective method that allows for simultaneous screening of many potential binders [276]. This approach has been used to generate human single‐chain Abs (scFvs) against mannotriose [277]. The primary obstacles of this method are the stability of the Ab fragments and their relatively lower avidity due to their monovalent binding compared with canonical bivalent Abs. Other display methods, such as bacterial and yeast display, work in a similar manner, but have not been applied to anti‐glycan Ab discovery.

In recent years, high‐throughput methods have been developed to generate mAbs from humans [278], mice [279], and other animal species. Especially relevant are approaches based on single B cell sorting and cloning [278, 280]. Typically, the antigen, such as native CPS, is fluorescently labeled and used as a bait to sort out antigen‐specific B cells from the spleen or blood via fluorescent activated cell sorting (FACS) or similar technologies (Figure 4C). Sorting without a bait is used to investigate the epitopes of specific B cell subtypes. The RNA sequences of single cells, encoding heavy and light chain genes, are converted to complementary DNA via reverse transcription–polymerase chain reaction (RT‐PCR) and Ab‐genes are amplified via PCR. Respective sequences are used to clone and produce antigen‐specific mAbs. Another approach relies on the culture of single Ab‐secreting memory B cells isolated from patients and grown in vitro, followed by the identification of antigen‐specific Abs in the supernatant, cloning, and mAb production [281]. Similar methods have continuously been improved and accelerated [282].

Isolation of Abs from other animals is advantageous for developing antibodies of different formats and species. For instance, immunization of other mammals, such as camelids, can be used to produce heavy‐chain‐only Abs that can in turn be engineered to produce nanobodies (Figure 4D) [283]. Nanobodies can be selected through additional display methods or mass spectrometry, and isolated with FACS, magnetic‐activated cell sorting (MACS), or chromatographic methods [284]. Alternatively, they can be directly isolated from serum through a modified affinity chromatography approach followed by liquid chromatography–mass spectrometry (LC‐MS/MS) of the fractionated variable domains and de novo sequencing [285].

The hybridoma technology and other advanced methods have generated a vast set of anti‐glycan‐Abs. Several of these anti‐glycan‐Abs have moved into clinical development [286]. In this chapter, we focus on the technologies and Abs that have been generated or characterized with the help of synthetic glycans.

#### 5.2.1 Synthetic Glycans for the Generation of mAbs Against Bacterial Glycans

Synthetic bacterial glycans hold great promise for the development of antibacterial therapeutic and diagnostic tools. However, the full potential of bacterial glycans is hindered by the complexity of their synthesis and the limited availability of building blocks. Using defined synthetic glycans to immunize animals and subsequently generate antibodies is an attractive approach to generate protective antibodies directly against the target epitope.

Two mAbs against the lipoarabinomannan) LAM (structures from Mtb were generated by immunizing rabbits with synthetic LAM‐related polyarabinose oligosaccharide fragments containing repeating units of arabinose subunits coupled to BSA. LAM‐targeting mAbs were then characterized using synthetic glycan microarrays [287].

Mice immunized with a glycoconjugate composed of a synthetic analog of *C. difficile* surface polysaccharide‐I (PS‐I) coupled to CRM197, followed by mAb development through hybridoma technology gave rise to mAbs. Multivalent display of PS‐1 using a synthetic scaffold to mimic its display on the bacterial cell surface, initiated a sufficiently high immune response to generate mAbs that bound the synthetic glycan with nanomolar affinity [288].

Synthetic analogues can resemble larger structures and result in multivalent epitopes. *Yersinia pestis* is the causative bacterial agent of plague. The inner core of the bacterial LPS is composed of three heptose units that form the epitope (L‐α‐D‐Hep*p*‐(1→7)‐L‐α‐D‐Hep*p‐*(1→3)‐L‐α‐D‐ Hep*p*). LPS‐specific mAbs were generated by immunizing mice with the semisynthetic trimer‐glycoconjugates followed by mAb development [289]. The interactions between the synthetic LPS‐trimer and the anti‐LPS mAbs were mapped by combining glycan microarray screening, surface plasmon resonance (SPR), and saturation transfer difference nuclear magnetic resonance (STD NMR) [274].

The structural diversity of bacterial glycan epitopes enables the development of highly specific mAbs that target harmful bacterial species and serotypes, while sparing those that are beneficial to human health, but limits the use of mAb therapy to provide broader antibacterial protection. Extensive efforts focus on developing Abs targeting more conserved structures on the bacterial surface to offer broader protection. Such an example is a murine mAb generated against synthetic glycerol phosphate (GroP)‐based teichoic acids (TA), which are bacterial cell wall components shared by different Gram‐positive bacteria such as *Enterococcus* and *Staphylococcus* species. The binding mechanism between the mAb and its TA target was characterized by glycan microarrays, SPR, ELISA, and STD‐NMR spectroscopy. The authors demonstrated that the number and chirality of the GroP residues, as well as an indirect contribution from a glucose subunit, are essential for Ab binding [290].

Notably, synthetic glycan microarrays serve as an excellent tool to characterize the binding epitope of antibodies generated by immunizing mice with native structures, often combined with additional methods. Synthetic oligosaccharides were used to determine the epitope of mAb C8 targeting *A. baumannii* 17978. Synthetic glycan microarrays helped to establish a tetrasaccharide fragment as the binding epitope and revealed that the 2,3‐diacetamido‐4‐*O*‐acetyl glucopyranosyl uronate in the repeating unit was crucial for binding [72]. Similarly, the specificity of a humanized Ab [291] against the lipopolysaccharide O25b antigen of *E. coli* ST131‐O25b:H4 was established using synthetic glycan microarrays containing oligosaccharide antigens of different lengths and frameshifts. Nanomolar affinity values were determined toward synthetic structures containing two, three, or fifteen repeating units by SPR experiments [57].

Murine mAbs specific to LTA, a significant component of *C. difficile* cell wall, were developed using hybridoma technology. The thermal stability, solubility, and binding to native LTA by the mAbs were established by SPR and competitive ELISA. The ELISA with immobilized synthetic structures containing the LTA core unit or lipid region, revealed that the minimal epitope was composed of the trimer repeating unit of [(→6)‐α‐D‐GlcNAc‐(1→3)‐α‐D‐(6‐P→)‐GlcNAc‐(1→2)‐D‐GroA] [292].

Ab binding to WTA from the cell wall of *S. aureus* focused on two humanized mAbs, 4461 and 4497 to identify phosphate groups and ribitol phosphate units of the WTA backbone as contributing to the mAb binding glycan epitope. The study combined glycan microarray, NMR, and computational modeling unraveled the cross‐reactivity of mAb 4497 for β‐(1→3)/(1→4)‐GlcNAc‐modified WTA [293].

Mice immunized with isolated A band polysaccharide of *P. aeruginosa* generated three murine mAbs, 3C4 (IgG2b), 3B8 (IgM), and 1B1 (IgM). Synthetic glycans were then used in an inhibition ELISA and in SPR experiments to further characterize the binding epitopes. Interestingly, 1B1 demonstrated binding toward all 3‐O‐methyl rhamnan tri‐, tetra‐, and penta‐saccharides with micromolar affinities, and was able to bind the majority of tested clinical *P. aeruginosa* isolates [294].

To date, synthetic glycans have not been used to generate human mAbs directly. However, synthetic glycan microarrays have helped to determine the binding patterns of human anti‐glycan Abs. Human mAbs against Mtb‐related surface glycans by a single B cell sorting and cloning approach bound the native surface polysaccharide AM and related glycolipid LAM that were used as a bait to sort out glycan‐specific B cells. Glycan microarrays with synthetic structures were used to determine the exact binding epitopes of the mAbs to be Man_3_Ara_4_, the related branched (Man_3_)_2_Ara_6_, and to terminal arabinan motifs. Two mAbs recognized the target structures with affinities in the mid‐nanomolar range [295]. In a high‐throughput manner, it was shown how a single B cell approach using blood and bone marrow samples from human subjects leads to the production of 516 human mAbs including 26 anti‐glycan mAbs. The primary targets were microbial carbohydrates, and the Abs primarily originated from IgG+ memory B cells, a promising source of anti‐glycan Abs [148]. Several novel high affinity mAbs were identified. Synthetic glycan microarrays helped to fine‐map the binding epitope of a human IgA mAb derived from single B cells in the cerebrospinal fluid of multiple sclerosis patients. The Ab did not bind self‐antigens, but rather microbial glycans from *E. coli* and *S. pneumoniae* containing *N*‐acetyl mannosamine [296].

#### 5.2.2 Synthetic Glycans for the Generation of mAbs Against Fungal Glycans

Anti‐glycan monoclonal antibodies targeting the glycan‐rich fungal cell wall are attractive targets for the development of potential therapeutics. mAbs against *Aspergillus fumigatus* galactomannan were prepared by hybridoma technology. Mice were immunized with a synthetic pentasaccharide β‐D‐Gal*f*‐(1→5)‐[β‐D‐Gal*f*‐(1→5)]_3_‐α‐D‐Man*p*, conjugated to BSA. Two mAbs, 7B8 and 8G4, bound the pentasaccharide with nanomolar affinity. A glycan microarray containing related synthetic oligosaccharide fragments demonstrated that mAb 8G4 and mAb 7B8 recognized the pentasaccharide and a trisaccharide, respectively. The Abs did not cross‐react with other bacterial and fungal strains and may potentially be used to detect Aspergillosis infections [297]. BSA conjugates of β‐(1→3)‐glucans nonamers from the fungal cell wall and mice immunization were used to generate several mAbs. Glycan microarray analysis revealed linear tri‐, penta‐, and nonaglucosides, as well as branched octasaccharides as mAb 5H5 binding epitope. A second mAb, 3G11, recognized linear chains. The affinity constants were in the nanomolar range for both mAbs that bound yeasts and filamentous fungi, but not bacteria. 3G11 and 5H5 demonstrated protective activity in an in vivo mouse model of systemic candidiasis [270]. Synthetic glucuronoxylomannan (GXM) structures of *C. neoformans* serotype A and D were employed to determine the binding patterns of seventeen mouse mAbs by glycan microarrays. A serotype A decasaccharide was identified as target of neutralizing mAbs [298]. A glycan microarray with 26 *C. neoformans*‐related glycans served to determine the binding pattern of 16 mAbs. Protective and non‐protective Abs shared a conserved reactivity to the M2 motif of GXM, but protective IgG Abs showed cross‐reactivity to at least two GXM motifs. The M2 hexasaccharide motif is characterized by an α‐mannan backbone, modified by β‐(1→2)‐xyloses (Xyl) on the first two mannoses and a β‐(1→2)‐glucuronic acid on the third mannose [299]. This study highlighted that the selection of epitopes is critical for the development of neutralizing mAbs.

The epitope specificity of two murine mAbs, CM532, and FG70, toward fungal mannan and β‐(1→3)‐glucans was studied. The CM532 Ab obtained by immunization with a pentamannoside KLH conjugate, bound the trisaccharide β‐Man‐(1→2)‐α‐Man‐(1→2)‐α‐Man epitope. In contrast, Ab FG70, obtained by immunization with a linear β‐(1→3)‐glucan heptamer conjugated to KLH, recognized a linear β‐(1→3)‐linked pentaglucoside fragment and epitopes containing additional 3,6 branches, underlining the need for careful synthetic antigen selection [300].

MAbs are often used in diagnostic tools for the detection of fungi in patient samples. However, their binding epitopes are often not well characterized. A novel murine mAb was generated by immunizing mice with fragmented cell wall components from *Aspergillus parasiticus*. The resulting mAb AP3 bound to several *Aspergillus* species, and microarray analysis revealed that AP3 recognizes oligo‐[β‐D‐Galf‐(1→5)] sequences containing four or more residues [301].

#### 5.2.3 Synthetic Glycans for the Generation of mAbs Against Cancer and Mammalian Glycans

Specific targeting of aberrant human or non‐human mammalian glycans on cancer cells using mAbs has emerged as a powerful tool in cancer theranostics and clinical oncology. The production of highly specific mAbs against the Tn antigen is challenging, but several groups have managed to generate mAbs against Tn or Tn‐MUC‐1 via synthetic means. SN‐101, an anti‐Tn‐MUC‐1 IgG1, is highly specific and selective binding to 20 relevant MUC‐1‐related compounds with affinity in the submicromolar range [302]. mAbs against SSEA‐4, a TACA expressed on multiple cancers [303] were produced by immunizing mice with a synthetic SSEA‐4 vaccine [304] and produced Abs via hybridoma technology. The SSEA‐4‐specific mAb was then humanized, and the best binder was selected [303].

Nanobodies targeting TACAs were produced using synthetic glycans. Due to their lower molecular weight, which enables better penetration into the dense tumor microenvironment, and improved stability as a stand‐alone antibody fragment or chimeric antigen receptor (CAR), nanobodies are advantageous for cancer diagnosis and therapy compared with canonical Abs. A synthetic Globo‐H carbohydrate was used to immunize an alpaca and generate glycan‐specific heavy‐chain Abs that were used for the production of Globo‐H‐specific nanobodies. The nanobody bound with relatively low binding affinity, but high specificity to synthetic and native glycans, and improved its avidity by covalent trimerization that improved its dissociation constant (K_D_) value nine fold [283].

Cell surface *N*‐glycans are involved in various biological processes in mammals and other biological entities, yet few Abs targeting *N*‐glycans are available. *N*‐glycan‐specific Abs from an array of five synthesized, representative, human *N*‐glycans conjugated to the Qβ carrier were used to generate *N*‐glycan targeting IgGs in mice, but glycan microarray and ELISA studies revealed that the Abs were directed mainly to the shared chitobiose core and were unspecific for the respective *N*‐glycan structures [305]. The *O*‐linked glycosylation of GalNAc to tyrosine, discovered in 2011, is largely unknown. A mAb that recognized GalNAc‐tyrosine with high affinity and specificity on glycan microarray bound to human tissue and cell lines [306].

Minor differences in monosaccharides or linkages in mammalian glycans can strongly affect mAb binding to the glycoepitope. Therefore, defining mAb specificities is crucial, and synthetic glycans are powerful tools for this purpose. Two mAbs: human IgG1 ReMab6 and murine IgM ReBaGs6, are specific for Tn glycopeptides. The Abs were characterized using a range of biological and biochemical approaches, including synthetic glycopeptide microarrays with immobilized Tn and Tn‐related structures. Both mAbs recognized di‐ and tri‐Tn on mucin‐derived glycopeptides, with minimal interaction with synthetic IgA1‐containing Tn glycopeptides. Enzymatic sialylation or galactosylation of the microarray to generate sTn or TF antigens, respectively, abolished ReMab6 binding, demonstrating its high specificity for the Tn antigen [160].

Two studies used a neoglicolipid‐based glycan/glycopeptide microarray and complementary methods to investigate the specificity and selectivity of the preclinical anti‐sTn L2A5 mAb [307, 308]. Characterization of L2A5 mAb used glycan microarrays with up to 475 different structures. L2A5 was specific for sTn and some other sialylated TACAs, including the α‐(2→6)‐linked sialyl core‐1 [307].

Similarly, the minimal epitopes of two novel Abs, R‐6C and R‐13E, raised against pluripotent stem cells were determined. ELISA and an NGL glycan microarray with immobilized synthetic glycans revealed that the minimum epitopes of those Abs were sialylated keratan sulfate glycosaminoglycans and lacto‐*N*‐fucopentaose I structures. These Abs are valuable for studies on stem cells, cancer, and regenerative medicine [309].

A collection of 41 linear and branched poly‐LacNAc glycans representative of the rare blood group I‐antigen, characterized by branched poly‐LacNac chains, were explored. The structures are associated with various pathophysiological processes that remain poorly understood. The researchers used microarrays to study the binding patterns of three human‐isolated anti‐i/I mAbs and other GBPs. Their findings revealed unique recognitions of I‐branches, as well as a strong binding to oxidized forms of linear poly‐LacNAc, providing valuable insights into their functions and novel therapeutic avenues [144].

Modified synthetic glycans on arrays can be used to investigate Ab binding specificity, but also selectivity. The differential selectivity and cross‐reactivity of GBPs toward fluorinated analogues of the Lewis^X^ TACA as a model glycan were studied. Deoxy‐fluorination may provide enhanced and more specific interactions between proteins recognizing the same glycan. A library of 150 site‐specific fluorinated Lewis^X^ glycoforms was synthesized enzymatically and used to construct a neoglycolipid‐based microarray with a subset of 24 mono‐ and poly‐fluorinated Lewis^X^ structures. The different specificities of Lewis^X^‐binding proteins, including Abs were evaluated. Fluorination increased the binding to certain Lewis^X^ fluorinated analogues while reducing the binding to others, thereby enhancing the selectivity of the binders [310]. Similarly, sialylation and sulfation affect molecular recognition. Sulfogangliosides are sulfated derivatives of inherently sialylated gangliosides and play a role in various biological processes. A set of 21 sulfated and sialylated ganglio‐oligosaccharides was chemoenzymatically synthesized and printed on glycan microarrays to investigate the binding of GBPs, including anti‐ganglioside Abs against GM1 and GD1a. The anti‐GM1 and anti‐GD1a mAbs specifically bound to GM1 and GD1a oligosaccharide, respectively, and 6‐O‐sulfation on GalNAc abolished binding of the anti‐GM1 Ab while increased binding of the anti‐GD1a Ab [130]. Glycan modifications can be used to fine‐tune mAb recognition and binding.

#### 5.2.4 Recent and Ongoing Clinical Trials

MAbs developed using synthetic glycans are under investigation in ongoing clinical trials (Table 3). A recent study investigated the binding specificities of a human mAb F598 against PNAG, found on the surface of various bacteria and fungi [311]. The mAb was generated using the hybridoma technology from blood‐derived human B cells. MAb F598 bound highly acetylated structures containing three to four acetylation sites. The acetylation position was essential for mAb binding [220]. A Phase II clinical trial with the anti‐PNAG mAb F598, was recently terminated, and the study design is being reevaluated (NCT03222401).

**TABLE 3:** mAbs produced using synthetic and semi‐synthetic glycans.

mAb	Isotype	Target	Manufacturer	Clinical trial	Phase
F598 IgG2	Human IgG2	PNAG	Alopexx Pharmaceuticals	NCT03222401	II
MVT‐5873 (HuMab‐5B1)	Human IgG1	Sialyl‐Lewis^A^ (CA19‐9)	BioNTech	NCT02672917	I
MVT‐1075 (^177^Lu MVT‐5873)/MVT‐5873	Human IgG1	Sialyl‐Lewis^A^ (CA19‐9)	BioNTech	NCT03118349	I
MVT‐2163 (^89^Zr MVT‐5873)/MVT‐5873	Human IgG1	Sialyl‐Lewis^A^ (CA19‐9)	BioNTech	NCT02687230	I
MVT‐2163 (^89^Zr MVT‐5873)/MVT‐5873	Human IgG1	Sialyl‐Lewis^A^ (CA19‐9)	Memorial Sloan Kettering Cancer Center	NCT04883775	I
MVT‐5873	Human IgG1	Sialyl‐Lewis^A^ (CA19‐9)	National Cancer Institute	NCT03801915	II
OBI‐888	Humanized IgG1	Globo‐H	OBI Pharma	NCT03573544	I/II
OBI‐999 (monomethyl auristatin‐OBI‐888)	Humanized IgG1 ADC	Globo‐H	OBI Pharma	NCT04084366	I/II
OBI‐R007	CAR‐T	Globo‐H	OBI Pharma	—	—

Since the first publication of the B72.3 mAb targeting the TACA sTn [312], extensive efforts have been made to develop clinically approved anti‐glycan Abs for cancer diagnosis and therapy. A clinical‐stage mAb derived from a synthetic glycan vaccine conjugate is *MVT‐5873*. *MVT‐5873* (HuMab‐5B1) is a fully human IgG1 that targets sialyl‐Lewis^A^, isolated from blood lymphocytes of a breast cancer patient immunized with synthetic sialyl‐Lewis^A^ KLH conjugate [273, 313, 314]. BioNTech initially evaluated the safety and tolerability of *MVT‐5873*, combined with chemotherapy, in a Phase I study (NCT02672917) in subjects with CA19‐9‐positive pancreatic or other cancers. However, the study was terminated for unspecified reasons. Shortly after, *MVT‐5873* was radiolabeled with lutetium ‐177 (^177^Lu) to form *MVT‐1075*. A Phase I trial (NCT03118349) tested *MVT‐1075* in combination with a blocking dose of *MVT‐5873* for targeted radioimmunotherapy of patients with previously treated CA19‐9‐positive cancers [315]. The study was terminated without published results. Similarly, another Phase I trial evaluated the zirconium ‐89 (^89^Zr) radiolabeled *MVT‐5873* (*MVT‐2163*) with and without *MVT‐5873* for positron emission tomography (PET) imaging (NCT02687230) in patients with pancreatic cancer or other tumors expressing CA19‐9. The Abs were well tolerated at all doses and demonstrated satisfactory results [316, 317, 318]. While this study was terminated, its promising results led to another Phase I clinical trial (NCT04883775) by MSKCC, which aimed to evaluate the attachment of *MVT‐2163* to pancreatic tumors and its potential for PET/ computed tomography (CT) imaging. The study was completed, but its results have not been published to date. The National Cancer Institute also investigated *MVT‐5873* in a Phase II clinical trial (NCT03801915) for the treatment of CA19‐9‐positive pancreatic cancer, cholangiocarcinoma, and metastatic colorectal cancer, administered before and after therapy [314]. The study was completed in 2023, but results have not been published yet.

OBI Pharma developed OBI‐888, a humanized mAb targeting Globo‐H. This mAb demonstrated promising results in vitro and in vivo, progressing to Phase I/II clinical trials (NCT03573544) for the treatment of locally advanced or metastatic solid tumors [319]. The mAb exhibited a favorable safety profile with minimal side effects; however, its limited anti‐tumor activity led to the termination of its development [319]. The promise of OBI‐888 progressed into a Globo‐H ‐specific antibody‐drug conjugate (ADC) (OBI‐999) and a CAR (OBI‐R007) for anti‐Globo‐H CAR‐T cell therapy. OBI‐999 (i.e., OBI‐888 with monomethyl auristatin cytotoxic payload) showed tumor growth inhibition in several cancer models and reached Phase I/II clinical trials (NCT04084366) for the treatment of multiple advanced solid tumors expressing Globo‐H. Despite a favorable safety profile with minimal side effects, the Phase II trial was discontinued as the company shifted focus to other anti‐cancer therapeutic candidates [320, 321]. Regarding OBI‐R007, although limited data is available, a robust and specific antitumor activity in Globo‐H‐expressing tumors was reported [322].

This research underlines the importance of synthetic glycans for the development of effective mAbs targeting pathogens and cancer, to pave the way to advance antibody‐derived therapies.

## 6 Summary and Outlook

Anti‐glycan antibodies are important in health, disease, and immunosurveillance. Distinct glycan structures are recognized by the immune system, induce an immune response and the formation of anti‐glycan antibodies. Synthetic strategies enable the generation of pure oligosaccharide epitopes with defined stereochemistry and connectivity. Efforts using novel synthetic methods such as automated glycan assembly and chemoenzymatic synthesis have lowered synthesis cost and time, enabling the generation of hundreds of human and microbial glycan epitopes. Recent improvements in protecting group strategies, the availability of universal building blocks, and integration with enzymatic synthesis will ensure improved synthetic glycan availability.

Diverse sets of complex synthetic glycans and glycopeptides have helped to elucidate structure–function relationships and the biological role of distinct glycan structures in health and disease. Synthetic glycan microarrays enable the investigation of anti‐glycan antibody repertoires in a high‐throughput and high‐content manner, enhancing our understanding of their role and expanding the search for novel biomarkers for early disease detection and patient stratification. In addition, glycan arrays enable epitope mapping of existing mAbs, and efficient design of glycoconjugate vaccines.

Synthetic glycan‐based vaccines offer a promising therapeutic solution for microbial infections and cancer. Recent developments demonstrated the critical role of carrier proteins, proper adjuvants, controlled epitope density, and multivalent antigen presentation for the efficacy of semi‐ and fully synthetic glycoconjugate vaccines. Despite the challenges associated with generating high quality mAbs against glycan epitopes, the increased availability of synthetic glycans and novel approaches based on next generation sequencing has the potential to increase the number of available mAbs and improve their characterization. Advances in omics methods, such as glycomics, glycoproteomics, and glycolipidomics, will facilitate a more comprehensive identification of cell‐surface glycoconjugates, offering an optimistic future for additional glycan‐based therapeutics. Emerging artificial intelligence (AI)‐based tools will enable personalized medicine by predicting altered cell‐surface glycans by integrating synthetic microarray results with glycomics, glycoproteomics, and single‐cell gene sequencing alongside patient‐derived data. As a result, profiling glycocalyx signatures and altered profiles of anti‐glycan antibodies in large cohorts will enable the establishment of novel criteria for disease detection and sub‐classification and unveil new definitions for patient stratification.

While synthetic glycans have revolutionized glycobiology, enabling the development of exciting tools for basic and translational research, a new era in glycan‐based biomedical and biotechnological applications will be driven by AI and rely on a multidisciplinary integration of clinical medicine with organic chemistry, immunology, mass‐spectrometry, and biomedical engineering, enabling the development of exciting anti glycan antibody‐based tools for basic and translational research.

## Author Contributions

**Fabienne Weber**: writing – original draft, investigation, visualization, data curation. **Aina Valenti**: writing – original draft, visualization, investigation, data curation. **Jiří Ledvinka**: writing – original draft, visualization, investigation, data curation. **Oren Moscovitz**: writing – review and editing, supervision, conceptualization, data curation, project administration. **Peter H. Seeberger**: writing – review and editing, conceptualization, supervision, resources, data curation, project administration, funding acquisition.

## Conflicts of Interest

The authors declare no conflicts of interest.

## Data Availability

Data sharing not applicable to this article as no datasets were generated or analyzed during the current study.
